# Expression Analysis of an Evolutionarily Conserved Alternative Splicing Factor, Sfrs10, in Age-Related Macular Degeneration

**DOI:** 10.1371/journal.pone.0075964

**Published:** 2013-09-30

**Authors:** Devi Krishna Priya Karunakaran, Abdul Rouf Banday, Qian Wu, Rahul Kanadia

**Affiliations:** 1 University of Connecticut, Physiology and Neurobiology, Storrs, Connecticut, United States of America; 2 University of Connecticut Health Center, Department of Pathology and Laboratory Medicine, Farmington, Connecticut, United States of America; Louisiana State University Health Sciences Center, United States of America

## Abstract

Age-related macular degeneration (AMD) is the most common cause of blindness in the elderly population. Hypoxic stress created in the micro-environment of the photoreceptors is thought to be the underlying cause that results in the pathophysiology of AMD. However, association of AMD with alternative splicing mediated gene regulation is not well explored. Alternative Splicing is one of the primary mechanisms in humans by which fewer protein coding genes are able to generate a vast proteome. Here, we investigated the expression of a known stress response gene and an alternative splicing factor called Serine-Arginine rich splicing factor 10 (Sfrs10). Sfrs10 is a member of the serine-arginine (SR) rich protein family and is 100% identical at the amino acid level in most mammals. Immunoblot analysis on retinal extracts from mouse, rat, and chicken showed a single immunoreactive band. Further, immunohistochemistry on adult mouse, rat and chicken retinae showed pan-retinal expression. However, SFRS10 was not detected in normal human retina but was observed as distinct nuclear speckles in AMD retinae. This is in agreement with previous reports that show Sfrs10 to be a stress response gene, which is upregulated under hypoxia. The difference in the expression of Sfrs10 between humans and lower mammals and the upregulation of SFRS10 in AMD is further reflected in the divergence of the promoter sequence between these species. Finally, SFRS10+ speckles were independent of the SC35+ SR protein speckles or the HSF1+ stress granules. In all, our data suggests that SFRS10 is upregulated and forms distinct stress-induced speckles and might be involved in AS of stress response genes in AMD.

## Introduction

Age-related Macular degeneration (AMD) is the leading cause of blindness in the aging population in the developed world. According to National Eye Institute, in 2010, approximately 2 million people were affected with AMD in the US and the number is estimated to go up to 3.6 million by 2030 (http://www.nei.nih.gov/eyedata/amd.asp). AMD affects the macula, the region in the retina that is responsible for sharp, central vision. AMD can be of two distinct forms: dry AMD (non-exudative or atrophic) or wet AMD (exudative or neo-vascular) of which dry AMD is the most common. AMD is a multifactorial disease that includes genetic, environmental and physiological components. The underlying cause of AMD is thought to be the hypoxic condition experienced by the photoreceptors leading to their degeneration [1]–[7]. Rod photoreceptors consume more O_2_ per gram of tissue weight than any other cell in the body [8]. This constant high energy demand makes the photoreceptors more susceptible to hypoxic stress. Factors such as oxidative stress, accumulation of autoxidative lipofuscin in the lysosomes of retinal pigmented epithelial (RPE) cells [9]–[14] and accumulation of drusen in between Bruch's membrane and the epithelial layer [15], [16] affect the RPE which results in the senescence of these cells. RPE not only plays a vital role in supplying nutrients and oxygen from choroidal vasculature to the photoreceptor cells, but also in removing the metabolic wastes from the photoreceptors. Since the outer segments of the photoreceptors interact with RPE, senescence of the latter affects the normal functioning of the former [17], [18]. Genome wide association studies have shown SNPs in genes including VegfA, VegfR2, Arms2, Htra1, and CFH to have significant association with AMD [19]–[25]. However, the role of alternative splicing in the pathogenesis of AMD is not well understood.

Alternative splicing is the process by which exons of protein coding genes are spliced in different combinations to produce multiple isoforms. Certain trans-acting factors called alternative splicing factors (ASFs) regulate the process of inclusion/exclusion of an exon in the final mRNA by either enhancing or repressing the recruitment of the spliceosome machinery. One group of ASFs that act as exonic splicing enhancers is SR proteins. These proteins are a family of highly conserved RNA binding proteins [26], some of which, like SF2/ASF and SC35, are also involved in constitutive splicing [27]–[30]. Typically, SR proteins contain one or two RNA recognition motifs (RRM) at the N-terminus and a serine-arginine (SR) rich domain at the C-terminus [26]. While the RRM helps the SR proteins to bind to their target RNA, phosphorylation status of serine and arginine in the RS domain determines the sub-cellular localization of these proteins. Since most SR proteins are known to shuttle between nucleus and cytoplasm, they have also been shown to play various other roles such as in mRNA export, mRNA turnover, mRNA stabilization and translation [31]–[33]. Members of SR protein family such as SFRS1 have been shown to play a role in the pathogenesis of AMD. Phosphorylated SFRS1 was shown to promote proximal site selection in exon 8 of VEGF to generate the angiogenic isoform, VEGF (165) in AMD [34], [35]. Our study focuses on another member of the SR family called Serine-Arginine rich splicing factor 10 (SFRS10), a known stress response gene.

SFRS10, also known as Tra2 beta, was originally identified as a stress-response gene named RA301. It was shown to be upregulated in cultured astrocytes during hypoxia followed by reoxygenation [36]. This was the first report of an ASF linked to stress-response mechanism. Other evidence of stress induced upregulation of Sfrs10 was shown by Tsukamoto *et al* [37] where it was observed that middle carotid artery occlusion caused upregulation of Sfrs10 in vascular smooth cells. Moreover, Sfrs10 upregulation has been reported in various other stress and/or disease conditions such as silicosis, arteriosclerosis, nerve injury, and breast cancer [37]–[41]. Besides upregulation, oxidative stress-induced translocation of Sfrs10 into the cytoplasm from its normal nuclear localization has also been reported [42]. In all, Sfrs10 is an ASF that has been shown to respond to episodes of hypoxic/oxidative stress and so we investigated the expression of SFRS10 in AMD retinae.

In this report, we show that while Sfrs10 shows pan-retinal expression in mouse, rat, and chicken, it is not observed in normal human retinae. In contrast, SFRS10 is upregulated in AMD retinae, which is in agreement with its previously described role as a stress response gene [36]. The varied expression of SFRS10 in different species and the upregulation of SFRS10 in AMD retinae are corroborated by the difference in the promoter of Sfrs10 in these species. Furthermore, Sfrs10 was seen in nuclear speckles that were independent of SC35 or HSF1. This data suggests a unique role for SFRS10 where it forms novel, sub-nuclear stress-induced structures that might be needed for AS of gene(s) responding to the hypoxic stress in AMD. Future investigation of the targets of Sfrs10 and their splice pattern shifts in disease states could shed light on the role of alternative splicing in the pathogenesis of degenerative disease like AMD and others.

## Materials and Methods

### Ethics statement

All procedures with the animals (mouse, rat, chicken, zebrafish) were performed in accordance with the animal protocol approved by Institutional Animal Care and Use Committee at the University of Connecticut (Permit number: A10-025). All animal procedures were performed so as to minimize suffering. Animals (mice and rats) were anesthetized using isoflurane followed by euthanasia by cervical dislocation. Zebrafish were sacrificed using overdose of tricaine methane sulfonate (MS222, 200–300 mg/l) by prolonged immersion.

### Animal procedure

The CD1 or the ICR mice from Charles River Laboratory, MA, were employed for mouse experiments. Chicken eyes were also obtained from Charles River Laboratory, Storrs, CT. The rat strain used was Wistar and was purchased from Charles River Laboratory. Zebrafish were obtained from Dr. Sylvain De Guise's laboratory at the University of Connecticut, Storrs, CT.

### Human samples

Slides with human retinal sections were obtained from Abcam, MA (http://www.abcam.com/) and from the National Disease Research Interchange (NDRI) (http://ndriresource.org/). In both cases, the authors were not involved in the procurement of the samples. Preprocessed de-identified retinal sections with the associated diagnosis were obtained for IHC analysis. Therefore, in this case the authors were issued a waiver from the University of Connecticut Institutional Review Board for human subject research. Briefly, the case history for all the samples was provided by NDRI. Also, to classify the AMD samples from the normal, pathological diagnosis was performed by Dr. Federico Gonzalez-Fernandez, MD, PhD, who collaborates with NDRI for the ophthalmologic pathology project. The parameters used in the diagnosis of AMD include accumulation of soft drusen beneath the retinal pigmented epithelium, disciform scar formation indicated by the fragmentation of Bruch's membrane and geographic atrophy.

In regards to the one sample (sample #7) obtained from the University of Connecticut Health Center (UCHC), consent from the next of kin, where applicable was obtained prior to post-mortem retinal tissue procurement. This protocol was approved by the University of Connecticut institutional review board for human subject research. The sample was de-identified before it was further processed in Dr. Kanadia's laboratory for IHC analysis and was conducted in compliance with the Health Insurance Portability and Accountability Act.

### Immunoblot

Retinal tissue extracts from mouse, rat, chicken and zebrafish were prepared in RIPA (50 mM Tris (pH 8.0), 150 mM Sodium chloride, 1% Igepal, 0.5% SDS) buffer containing 1× protease inhibitor cocktail (cOmplete mini, EDTA-free, Roche Diagnostics). Following the protein estimation of the extracts, 50 µg of protein was resolved on 4–20% Tris-glycine gradient gel (Invitrogen). The gel was transferred to a positively charged nylon membrane (Invitrogen), which was then subjected to immunoblot analysis as described previously [43]. Rabbit anti-Sfrs10 (1∶1000; Fitzgerald Inc., Product # 70R-1420) was used to detect Sfrs10 in the extract. Mouse anti-Gapdh (1∶500; Sigma Aldrich, Product # G8795) was used as the loading control.

### Immunohistochemistry (IHC)

All experiments were performed on 16 µm cryosections except for sample #1 which was 5 µm paraffin section. The cryosections were first hydrated in phosphate-buffered saline (PBS, pH 7.4) and washed three times (5 minutes each at room temperature (RT)), followed by incubation with PBTS buffer (1X PBS with 0.1% triton-X 100, 0.2% BSA and 0.02% SDS) for an hour at RT. Primary antibody (rabbit anti-Sfrs10, 1∶750; mouse anti-SC35, 1∶300, Abcam, Product # ab11826; rat anti-HSF1, 1∶100, Abcam, Product # ab61382) was incubated in PBTS buffer overnight at 4°C. Sections were washed with PBTS buffer containing 4′, 6-diamidino-2phenylindole (DAPI) (Roche diagnostics) 10 times (15 minutes each at RT). Following the washes, secondary antibody (anti-rabbit antibody conjugated with Alexa488 (Product # 21206), anti-mouse and anti-rat antibodies conjugated with Alexa568 (Product # A10037, A11077 respectively) 1∶750, Invitrogen) was incubated in PBTS buffer overnight at 4°C. Sections were washed with PBTS buffer 7 times (15 minutes each at RT), rinsed with PBS and covered with Prolong gold anti-fade reagent (Invitrogen) and coverslip glass. Slides with paraffin sections were preheated at 60°C for 1 hour. Slides were then cooled down until paraffin solidified. It was followed by 2 rinses with xylene for 5 min each, 2 rinses with 100% ethanol for 5 min each, one wash with 75% ethanol for 10 minutes, one wash with 50% ethanol for 10 minutes, one wash with 25% ethanol for 10 minutes and 2 washes with PBS. Rest of the IHC protocol was followed as described earlier.

### Serial IHC

Here the aforementioned IHC protocol was employed with one modification. Upon completion of the nuclear antigen detection by the first antibody, (primary – rabbit anti- Sfrs10, 1∶750 and secondary – Alexa488, 1∶750), a 10′/RT incubation with 4% paraformaldehyde was conducted. Subsequently, the cytoplasmic antigen detection by the second antibody (primary – rabbit anti - red/green opsin, 1∶300, Millipore, Product # AB5405 and secondary – Alexa568, Invitrogen, 1∶750, Product # 11011) was performed.

### Image Acquisition and 3D reconstruction

Confocal fluorescence microscopy was performed using Leica SP2. Images were subsequently processed using IMARIS (Bitplane Inc., CA) and Adobe Photoshop CS4 (Adobe Systems Inc., CA).

### Bioinformatics tools and databases

Alignment analysis was carried out using ClustalW tool (http://www.ebi.ac.uk/Tools/msa/clustalw2/). Promoter regions were characterized using tools such as CpGPlot (http://emboss.bioinformatics.nl/cgi-bin/emboss/cpgplot) and CpG Island searcher (http://cpgislands.usc.edu/).

## Results

### Sfrs10 is 100% conserved in mammals

**Figure 1:**
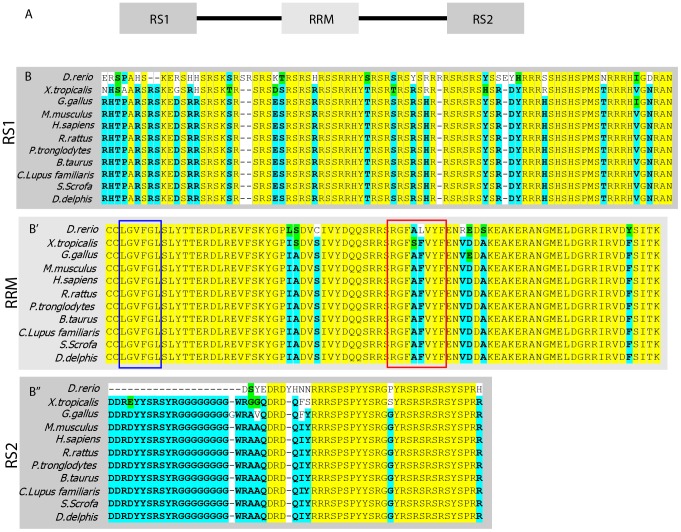
Sfrs10 is 100% conserved in mammals. A: Schematic of Sfrs10 protein with RRM (RNA recognition motif) flanked by two RS (Serine-Arginine dipeptide rich) domains. **B – B″**: Amino acid alignment of RS1 domain (B), RRM domain (B′), RS2 domain (B″) of Sfrs10 protein from different species.

Sfrs10 has three functional domains namely, N-terminal RS1 domain (31–113 amino acids (AA)), C-terminal RS2 domain (231–287 AA) and RNA recognition motif (118–196 AA) (Figure 1A). AA sequences corresponding to these functional domains were compared across different vertebrate species to assess the percentage of conservation of Sfrs10. The selected species were *Danio rerio* (zebrafish), *Xenopus tropicalis* (frog), *Gallus gallus* (chicken), *Pan tronglodytes* (chimp), *Bos taurus* (cow), *Canis lupus familiaris* (dog), *Homo sapiens* (humans), *Sus scrofa* (pig), *Mus musculus* (mouse), *Rattus rattus* (rat) and *Delphis delphis* (dolphin). The selection was based on the availability of the entire AA sequence in each class.

The entire RS1 domain was conserved 100% between chicken and all the mammalian species (yellow background, Figure 1B) while only the RS dipeptide region showed 100% conservation in all species (Figure 1B). RS2 domain was similar to RS1 domain in that there was 100% conservation in the RS dipeptide region among all the species (yellow background, Figure 1B″) with the entire domain conserved 100% among the mammals (Figure 1B″). Within the RRM of SR proteins there are two motifs called ribonucleoprotein 1 (RNP1), an octamer, and ribonucleoprotein 2 (RNP2), a hexamer, which are highly conserved [44]–[47]. In Sfrs10, RNP1 sequence is RGFAFVYF (159–166 AA) and RNP2 sequence is LGVFGL (125–130 AA). Comparison of RNP1 motif across the species showed 100% conservation except for zebrafish and frog, which had one AA change (phenylalanine to leucine in zebrafish, alanine to serine in frog) (red box, Figure 1B′). Similarly, comparison of RNP2 motif showed 100% conservation across all the species (blue box, Figure 1B′). Interestingly, the entire Sfrs10 AA sequence showed 100% conservation in mammals including the linker regions between the functional domains.

### Sfrs10 expression is conserved in mouse, rat and chicken retinae

**Figure 2:**
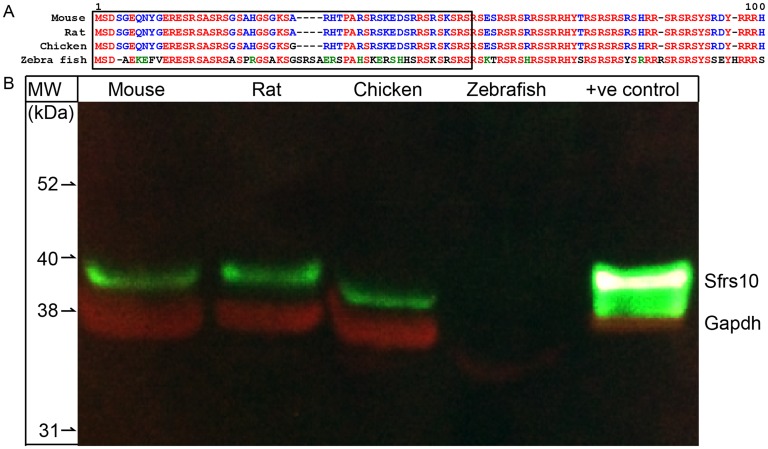
Expression of Sfrs10 is conserved in mouse, rat and chicken retina. A : Alignment of Sfrs10 amino acid (1–100) sequences from mouse, rat, chicken and zebrafish. The black box marks the region used as antigen to raise Sfrs10 antibodies. **B**: Immunoblot analysis for Sfrs10 (green) on retinal protein extracts from mouse, rat, chicken and zebrafish. The last lane has extract from HEK-293t cells that were expressing exogenous Sfrs10. Gapdh (red) serves as the loading control.

Given that Sfrs10 is highly conserved at the protein level across different species, we wanted to investigate whether its conservation was reflected in its expression. We chose mouse, rat, chicken, and zebrafish as these are widely used model organisms for retinal research. We employed immunoblot analysis using an antibody that recognizes the conserved N-terminal of the protein (black box, Figure 2A). A single band at the predicted (39 kDa) size was observed in mouse, rat, and chicken retinal extract (Figure 2B). This band was at the same molecular weight (MW) as the positive control, which was the protein extract prepared from HEK-293t cells transfected with a construct expressing full length Sfrs10 protein. Notably, no immunoreactivity was observed in the lane with zebrafish retinal extract (Figure 2B).

### Sfrs10 expression is pan-retinal in mouse, rat and chicken

**Figure 3:**
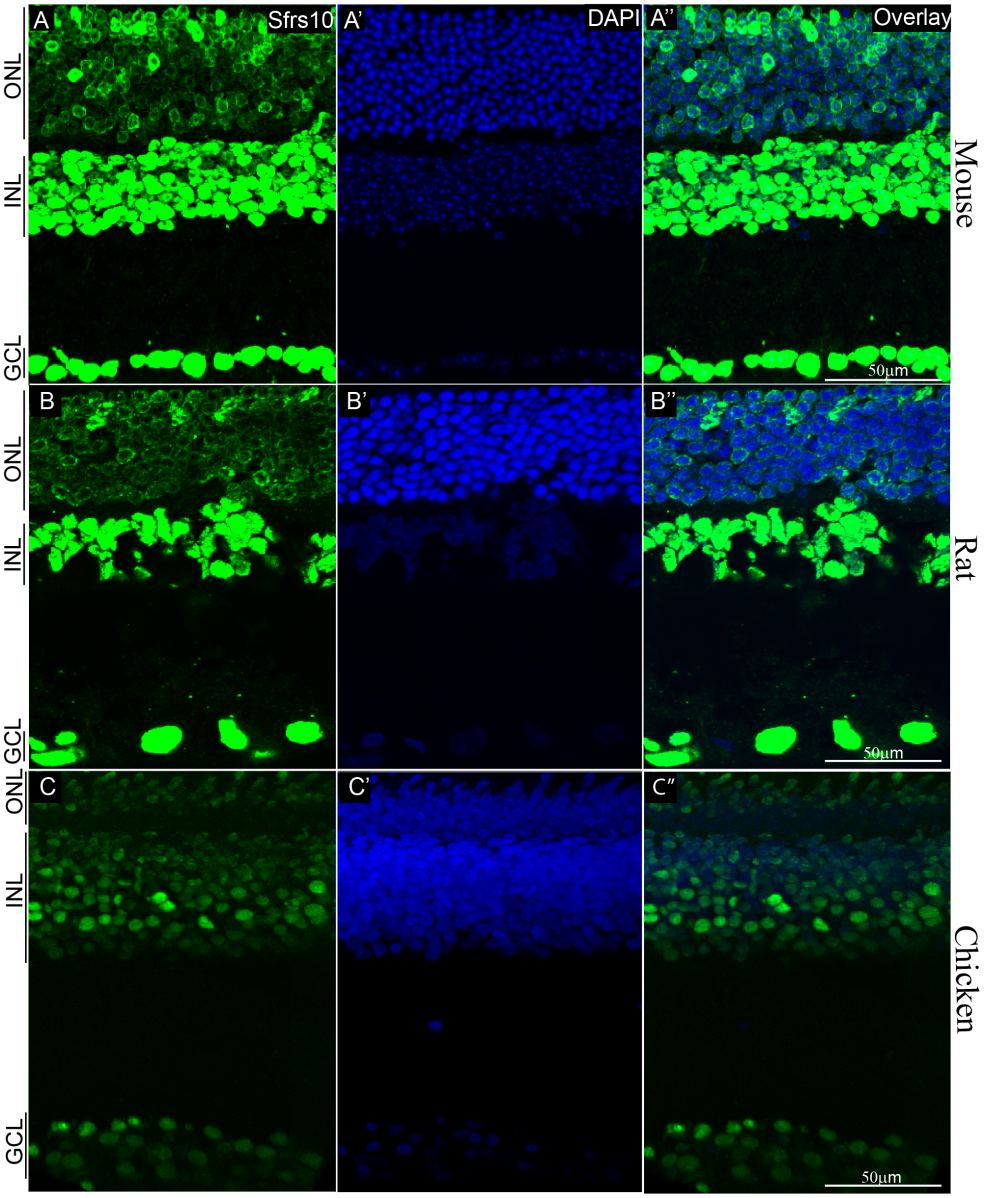
Sfrs10 expression is pan-retinal in mouse, rat and chicken. A–C″: IHC with rabbit anti-Sfrs10 (green) on retinal sections of adult mouse (A), adult rat (B) and adult chicken (C). DAPI (blue) marks all nuclei (A′, B′, C′). Scale Bar represents 50 µm.

To ascertain the cell type specific expression of Sfrs10, retinal sections from adult mouse, rat and chicken retina were subjected to IHC with the same antibody used for immunoblot analysis (black box, Figure 2A). In mouse, Sfrs10 was robustly expressed in all the cell types of the retina including, ganglion cells, amacrine cells, Müller glia, bipolar cells, horizontal cells and in both cone and rod photoreceptors. The expression pattern in photoreceptors was different with Sfrs10 expressed diffusely across the nucleus in cone photoreceptors, while the expression was peri-nuclear in rod photoreceptors (Figure 3A). In rat, Sfrs10 expression was similar to that of mouse and was observed in all cell types of the retina (Figure 3B). Similarly, in chicken, Sfrs10 was expressed in both the ganglion cell layer and in the inner nuclear layer, but in the photoreceptor layer there was a minor difference in the expression pattern. Sfrs10 expression was not observed in a subset of cells, which by the staining pattern was deduced to be rod photoreceptors (Figure 3C).

### SFRS10 is not expressed in normal human retinae but is upregulated in AMD retinae

**Figure 4:**
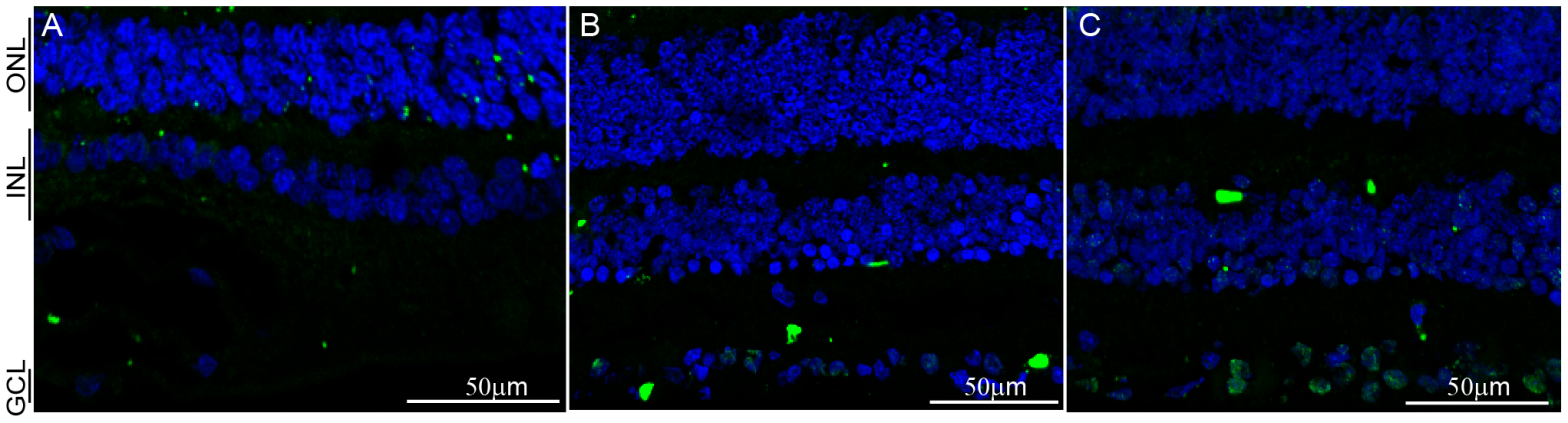
SFRS10 is not expressed in normal human retinae. A–C : IHC with rabbit anti-Sfrs10 (green) on sections obtained from individuals with normal retinae. DAPI (blue) marks all nuclei. Scale Bar represents 50 µm.

Given that Sfrs10 is a known stress response gene that is 100% conserved between mouse and human, we sought to investigate its expression in normal and AMD human retinae. For this, three independent normal retinal sections were subjected to IHC with anti-Sfrs10 antibody (Table 1). Sample #1 showed no Sfrs10 expression in any of the retinal layers (Figure 4A). Similarly, sample #2 showed no SFRS10 expression (Figure 4B). Again, sample #3 showed no SFRS10 expression, except for low levels in a few cells in the ganglion cell layer (Figure 4C).

**Table 1:** Case history of specimens analyzed.

Source	Sample No.	Age	Sex	Category	hours post mortem fix	Cause of Death	Medical History
Abcam	1	50	M	Normal	unknown	unknown	unknown
NDRI	2	74	F	Normal	4	COPD	no history available
NDRI	3	74	F	Normal	5	Cancer	Lung cancer, Pneumonia, Congestive heart failure
NDRI	4	88	F	AMD	7.8	Respiratory failure	Endometro cancer, osteoporosis, hypertension, glaucoma, macular degeneration, Hemangioma, aortic stenosis, insomnia
NDRI	5	86	F	AMD	7.4	Abcess	Macular degeneration, Diabetes, Cataract surgery, hemicholoectomy, hypertension, hypercholesterolemia
NDRI	6	89	M	AMD	6.5	CVA	Macular Degeneration, pneumonia, hypoxic respiratory failure, dementia
UCHC	7	76	F	unknown	4.0–5.0	Acute cardiac arrest	Unknown

Abbreviations: CVA – Cerebro Vascular Accident (Stroke); COPD – Chronic Obstructive Pulmonary Disease; NDRI – National Disease Research Interchange; UCHC – University of Connecticut Health Center.

**Figure 5:**
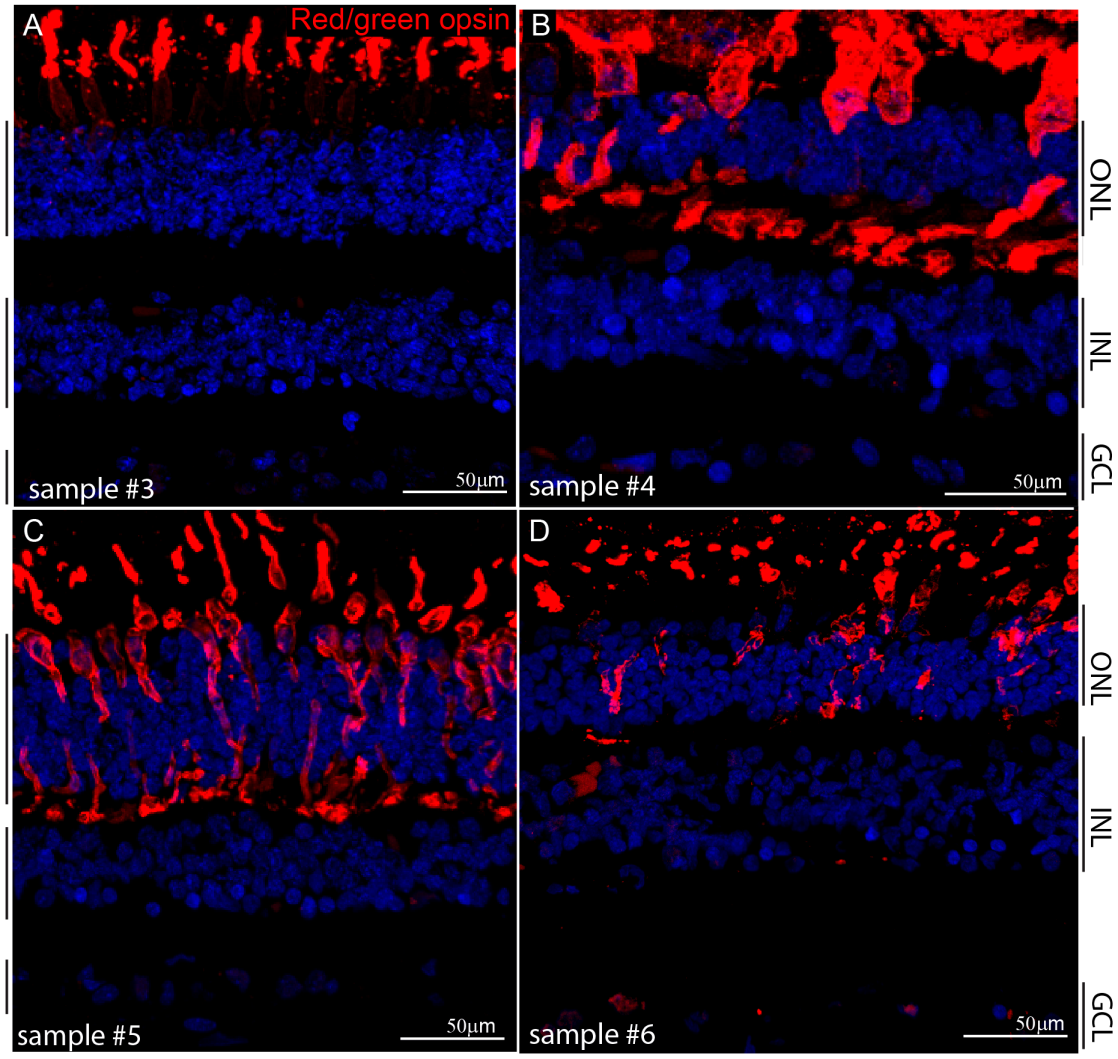
Red/green opsin is dispersed throughout the photoreceptor membrane in AMD. A–D: IHC with rabbit anti-red/green opsin (red) on sections obtained from individuals with normal (A) and AMD (B–D) retinae. DAPI (blue) marks all nuclei.

**Figure 6:**
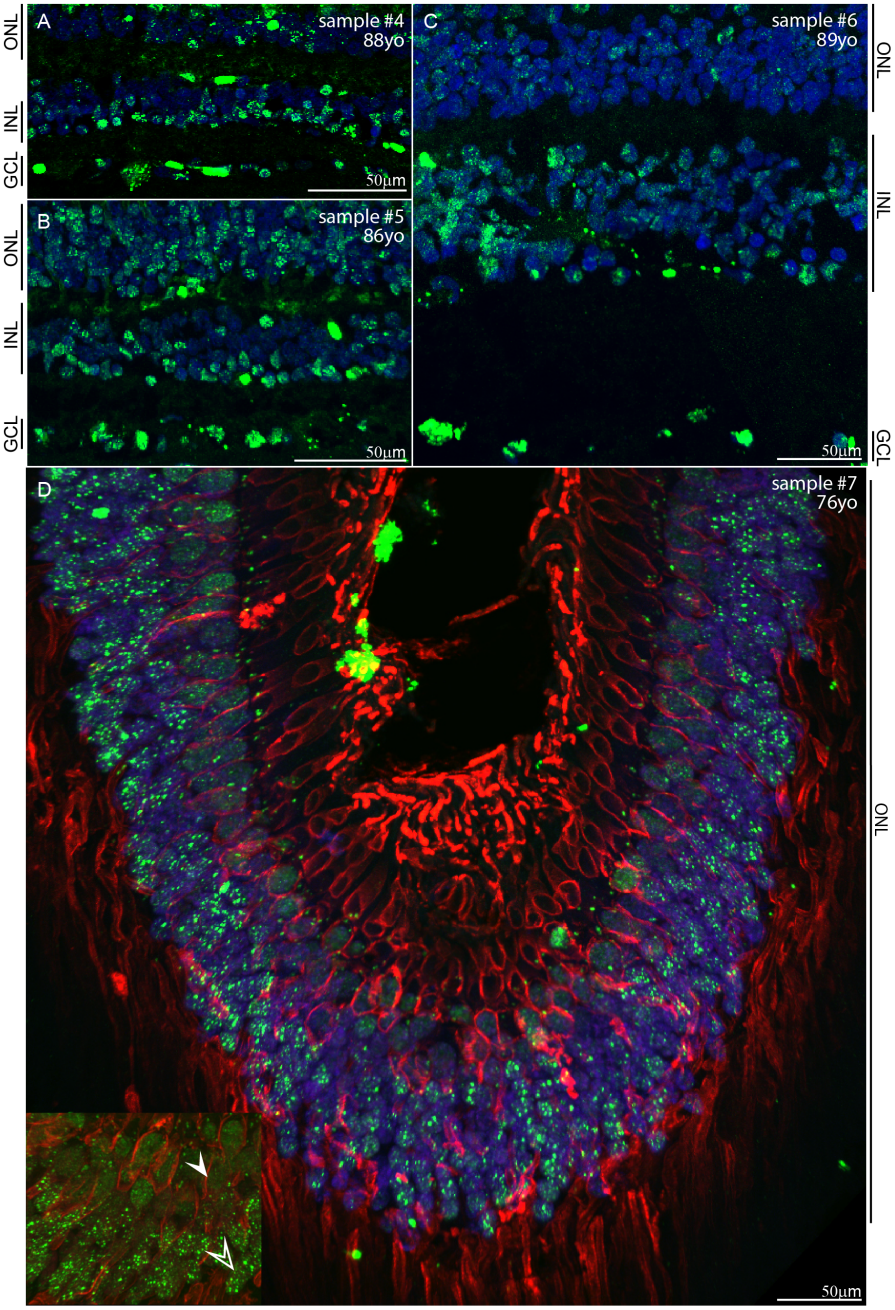
SFRS10 is upregulated in degenerating retina. A–C : IHC with rabbit anti-Sfrs10 (green) on sections obtained from individuals with AMD retinae. DAPI (blue) marks all nuclei. **D**: Serial IHC with rabbit anti-Sfrs10 (green) and rabbit anti-red/green opsin (red) on parafoveal section obtained from sample #7. DAPI (blue) marks all nuclei. Inset shows the expression of SFRS10 in the red and green cone photoreceptors indicated by solid white arrow and rod photoreceptors indicated by open white arrow. Scale Bar represents 50 µm.

Next, we wanted to investigate the expression in AMD, so we obtained three independent AMD samples from NDRI (Table 1). Here, the level of degeneration in the three samples was determined by the localization of L/M opsins in the cone photoreceptors by IHC. Under normal conditions, opsins are restricted to the membrane of the outer segments (OS) [48] as shown in normal sample #3 (Figure 5A). However, in case of the AMD retinae, L/M opsins were observed in the entire cone photoreceptor membrane (Figure 5B–D). This is in agreement with previous reports indicating that opsins in a degenerating retina relocalize to the entire photoreceptor membrane due to aberrant opsin trafficking [48]. Notably in Sample #4, vertical alignment of OS was lost and the thickness of the outer nuclear layer (ONL) was reduced compared to other two AMD retinae, indicating that this retina had a higher degree of degeneration (Figure 5B). Subsequently, all of these retinal sections were subjected to IHC with anti-Sfrs10 antibody. Sample #4 showed a distinct upregulation of SFRS10 in a speckled pattern (Figure 6A). Similar upregulation was seen in a few cells in all three nuclear layers in sample #5, where the layers were comparable to that in a normal retina (Figure 6B). The expression in sample #6 was similar to previous two samples with upregulation seen as distinct speckles in ganglion cells as well as in some cells in the INL and the ONL (Figure 6C). In addition, we analyzed the foveal/parafoveal regions from sample #7 by serial IHC. Again, the staining for red/green opsin showed that the opsin is redistributed throughout the membrane of the photoreceptor, indicating that the sample was undergoing degeneration (Figure 6D). In case of SFRS10, the staining in red and green cone photoreceptors (encapsulated by red/green opsin) was diffused throughout the nucleus (solid white arrows in the inset, Figure 6D), while, a speckled expression was observed in rod photoreceptors (open white arrow in the inset, Figure 6D).

### Human Sfrs10 promoter is different from that of rodents

**Figure 7:**
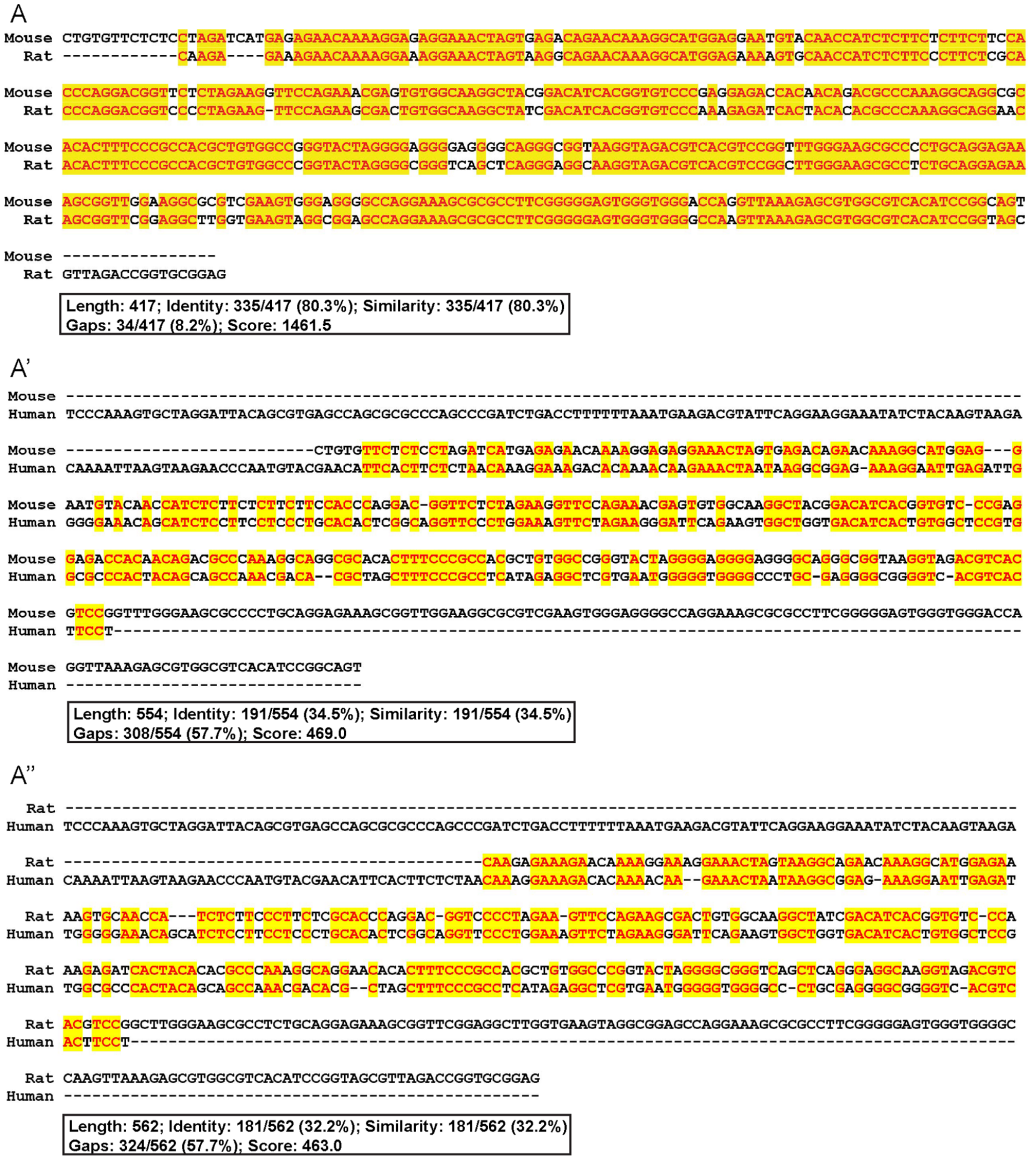
Comparitive analysis of mSfrs10 and hSfrs10 promoter region. A–A″: Comparison of Sfrs10 promoter in mouse vs. rat (A), mouse vs. human (A′), and rat vs. human (A″).

**Figure 8:**
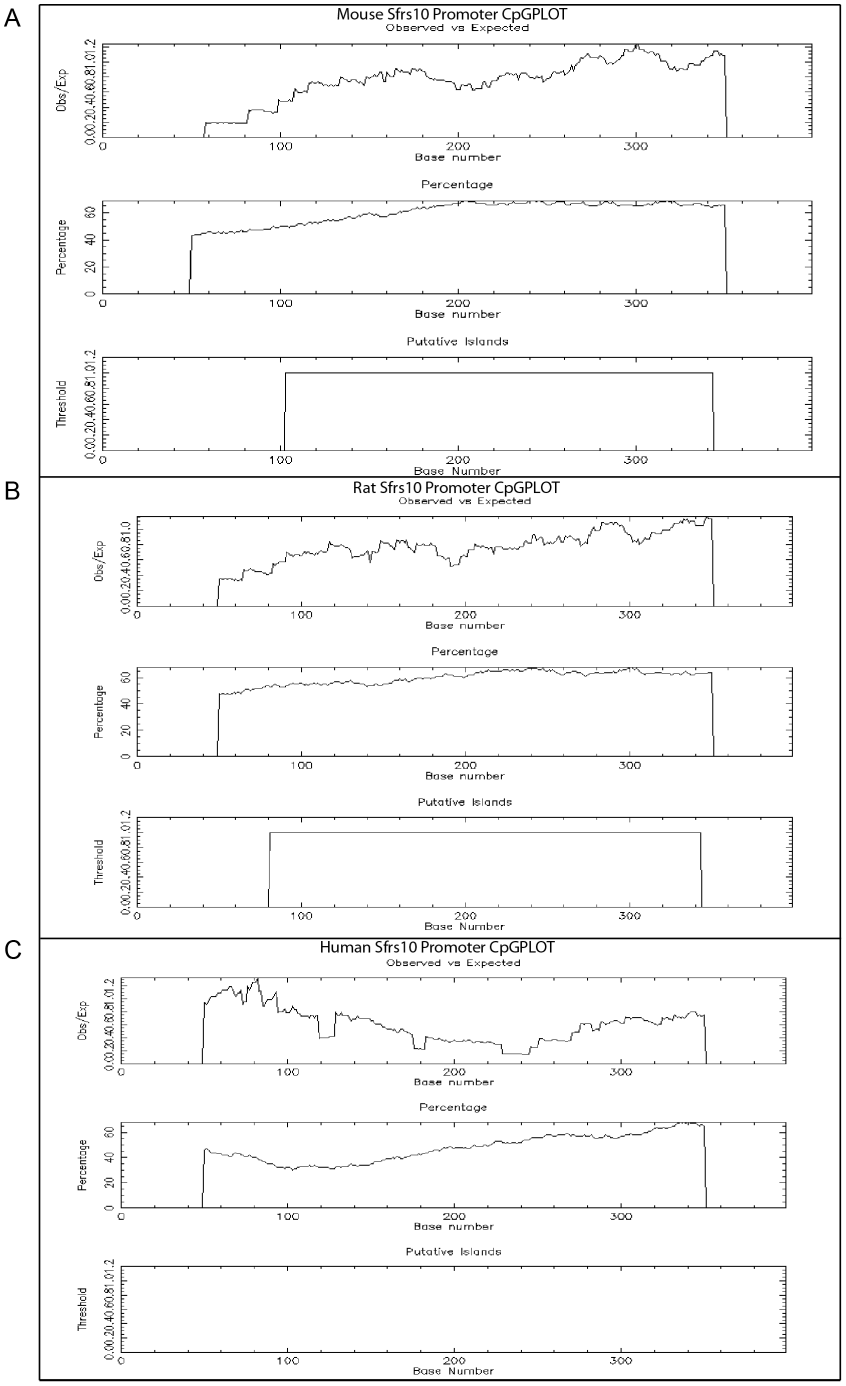
CpGPLOT analysis of Sfrs10 promoter region in mouse, rat and human Sfrs10. A–C: CPGPLOT generated by CpGPlot tool used for CpG island prediction. A, B – Mouse and rat Sfrs10 promoter region showed CpG islands; C – No CpG islands were seen in human Sfrs10 promoter region.

The lack of expression in normal human retina could be due to the difference in the transcriptional regulation of mouse and human Sfrs10. To investigate the Sfrs10 promoter region, multiple sequence alignment was performed on the 400 bp region upstream of transcription start site in human, mouse and rat (Figure 7A, A′, A″). The homology between mouse and rat was 80% (Figure 7A), but the homology between mouse and human promoter was 34% (Figure 7A′), and between rat and human promoter was 32% (Figure 7A″). Moreover, promoter in all three organisms did not have a conventional TATA sequence (Figure 7A, A′, A″). Since the mouse and rat promoters were highly similar and they both showed pan-retinal expression, we sought to investigate whether their promoters contained hallmarks of promoters associated with ubiquitously expressed genes. For this, the presence of GC-rich regions or CpG islands was investigated, as they are usually associated with “active” chromatin structures like those of house-keeping genes [49], [50]. Using CpG island detection tools such as CpG Island Searcher and CpGPlot, it was found that mouse and rat Sfrs10 promoter region had CpG islands (Figure 8A, B). These CpG islands had average GC content of 60–70% and observed/expected CpG ratio >60%. However, the human Sfrs10 promoter did not have CpG islands (Figure 8C).

### Sfrs10 forms stress-related speckles

**Figure 9:**
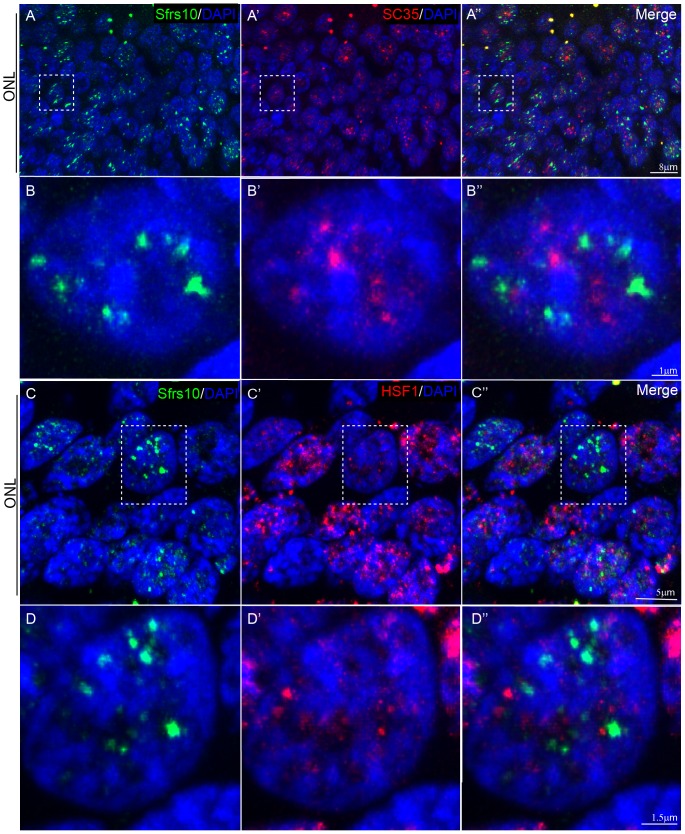
SFRS10 forms independent stress-related speckles. A–A″: IHC on human retinal section with anti-Sfrs10 (green), anti-SC35 (red). Nuclei are marked with DAPI (blue). **B–B″**: Magnified image of the boxed region in A–A″ showing non-overlapping Sfrs10+ speckles and SC35+ speckles. **C–C″**: IHC with anti-Sfrs10 (green), anti-HSF1 (red). Nuclei are marked with DAPI (blue). **D–D″**: Magnified image of the boxed region of C–C″ showing non-overlapping Sfrs10+ speckles and HSF+ stress granules.

Besides the upregulation of Sfrs10 in AMD retinae, it also showed a speckled pattern of expression. This pattern has been described for other SR proteins such as SC35 [51], [52]. In addition, Sfrs10 has been shown to colocalize with SC35 in human neuroblastoma cell lines [53]. Thus, human retinal sections were co-stained with Sfrs10 and SC-35, which showed that SFRS10+ speckles did not overlap with SC35+ speckles (Figure 9A–B″). This led us to investigate whether SFRS10+ speckles were indeed stress granules. HSF1 is known to form stress granules under various kinds of stress [54]. Also, HSF1 was shown to colocalize with SFRS10 in human colon cancer cell lines [55]. Therefore, AMD retinal sections were co-stained with Sfrs10 and HSF1, which showed that SFRS10+ speckles did not overlap with HSF1+ stress granules. Nonetheless, the presence of HSF1+ granules confirmed that this retina is under stress and that SFRS10+ speckles are distinct and stress induced (Figure 9C–D″).

## Discussion

### Sfrs10 is not detected in the zebrafish retina

Sfrs10 is a widely studied alternative splicing factor that is 100% conserved at the AA levels in most mammals. At the nucleotide level, Sfrs10 is one of the seven RNA-splicing associated genes that contain “exonic” class of “ultraconserved elements” [56]. This class of RNA-binding proteins is shown to auto-regulate their levels by the inclusion of a stop codon containing exon which is highly conserved in the vertebrate genome. This auto regulation is thought to be critical for the maintenance of cellular homeostasis of various classes of RNA-binding proteins. As Sfrs10 is one of the very few highly conserved genes, we investigated the expression of Sfrs10 in the retina of widely studied model organisms including, mouse, rat, chicken and zebrafish. Immunoblot analysis showed expression in mouse, rat and chicken, which suggests that its transcription is conserved along with its AA sequence. As predicted, no immunoreactivity was observed in the lane containing zebrafish retinal extract (Figure 2B). The antibody employed here was directed toward the N-terminus of Sfrs10, which is not conserved in zebrafish (black box, Figure 2A). Thus, the absence of the immunoreactivity could be failure of the antibody to recognize Sfrs10 and that it might still be expressed in the zebrafish retina.

### Sfrs10 is upregulated only in response to stress in human retinae

Interestingly, SFRS10 is 100% conserved at the AA level between mouse and human, yet it is not expressed normally in the human retina. This was surprising, but in agreement with its role as a stress response gene that would be normally suppressed. At the level of transcription regulation, CpG islands are shown to be associated with promoters of house-keeping genes and active genes by a comprehensive analysis of CpG islands in the European Molecular biology laboratory (EMBL) database [50], [57]. In all, the constitutive expression of Sfrs10 in mouse and rat retinae (Figure 3A, B) along with the presence of CpG islands (Figure 8A, B) suggests that it might regulate the AS of constitutively expressed genes in these organisms. In contrast, SFRS10 is not expressed in the normal human retinae (Figure 4A, B, C), and its promoter lacks CpG islands (Figure 8C), which suggests that SFRS10 might not be required for general maintenance. In contrast, it was upregulated in AMD retinae (Figure 6A, B, C), which is consistent with its role as a stress response gene. Interestingly, sample #7, for which there was no diagnosis of AMD, showed upregulation of SFRS10 suggesting that it was experiencing stress (Figure 6D). Since SFRS10 upregulation is often linked to hypoxic stress, one can extrapolate that retinal sample #7 was most likely undergoing hypoxic stress. In addition, the redistribution of red/green opsin staining throughout the membrane of photoreceptor (Figure 6D) indicates retinal degeneration [48].

In all, the upregulation of SFRS10 in AMD retinae suggests that it might be required for AS of a subset of genes involved in hypoxic stress response. For instance, a gene might normally be expressed but under stress conditions, it might undergo AS shift and the isoform responding to stress is regulated by SFRS10. Some of the known targets of Sfrs10 like Creb1, Pank2 are known to play a key role in metabolism [58], [59]. It could be that there is an increased demand for the isoform regulated by Sfrs10 under hypoxic stress. It is to be noted that both the aforementioned targets have isoforms in the retina (Data not shown). Future investigation involves testing of the splice pattern shifts in these targets under hypoxic stress.

### Sfrs10 does not co-localize with SC35 domain and is not part of stress-granules in human AMD retinae

Most SR proteins have been shown to be a part of the SC35 “nuclear speckle” under normal conditions. Presence of Sfrs10+ speckles independent of SC35 domain in AMD retinae (Figure 9A–B″) suggests that Sfrs10 and SC35 might not interact in the retina under hypoxic stress. It could be that SC35 regulates the splicing of genes that are normally required by the cell whereas Sfrs10 independently regulates a specific subset of genes that are required only under stress conditions. This partitioning of the SR proteins might provide efficient response to stresses such as hypoxia.

Non-overlapping Sfrs10 speckles with HSF1 stress granules in AMD retina (Figure 9C–D″) suggest that Sfrs10 does not interact with HSF1. It could also be that HSF1 regulates the transcription of genes that are required early in the stress response while Sfrs10 regulates the AS of the subset of genes that might be required later in the stress response. Overall, our data suggest that Sfrs10 might not be required for normal maintenance or functioning of neurons in human retina but is predominantly active under hypoxic stress, which is thought to be the underlying cause of AMD.
